# EasyDIVER: A Pipeline for Assembling and Counting High-Throughput Sequencing Data from In Vitro Evolution of Nucleic Acids or Peptides

**DOI:** 10.1007/s00239-020-09954-0

**Published:** 2020-06-11

**Authors:** Celia Blanco, Samuel Verbanic, Burckhard Seelig, Irene A. Chen

**Affiliations:** 1 Department of Chemistry and Biochemistry 9510, University of California, Santa Barbara, CA 93106 USA; 2 Department of Chemical and Biomolecular Engineering, University of California, Los Angeles, CA 90095 USA; 3 Program in Biomolecular Sciences and Engineering, University of California, Santa Barbara, CA 93106 USA; 4 Department of Biochemistry, Molecular Biology and Biophysics, University of Minnesota, Minneapolis, MN 55455 USA; 5 BioTechnology Institute, University of Minnesota, St. Paul, MN 55108 USA

**Keywords:** High-throughput sequencing, mRNA display, In vitro evolution, SELEX, Bioinformatics

## Abstract

In vitro evolution is a well-established technique for the discovery of functional RNA and peptides. Increasingly, these experiments are analyzed by high-throughput sequencing (HTS) for both scientific and engineering objectives, but computational analysis of HTS data, particularly for peptide selections, can present a barrier to entry for experimentalists. We introduce EasyDIVER (**Easy** pre-processing and **D**ereplication of **I**n **V**itro **E**volution **R**eads), a simple, user-friendly pipeline for processing high-throughput sequencing data from in vitro selections and directed evolution experiments. The pipeline takes as input raw, paired-end, demultiplexed Illumina read files. For each sample provided, EasyDIVER outputs a dereplicated list of unique nucleic acid and/or peptide sequences and their count reads.

**Electronic supplementary material:**

The online version of this article (10.1007/s00239-020-09954-0) contains supplementary material, which is available to authorized users.

## Introduction

In vitro evolution is a widely used method to isolate functional sequences with desired properties. These experiments, particularly RNA and DNA selections, are often analyzed by High-Throughput Sequencing (HTS) on the Illumina platform (Yokobayashi 2019; Blanco et al. 2019; Nguyen Quang et al. 2018). Increasingly, HTS analysis is being applied to peptide or protein selections, such as mRNA display. This technique is widely used to isolate functional peptides with desired properties. The selected mRNA-peptide fusions are reverse transcribed to cDNA and then prepared for sequencing by addition of specific adapter sequences to the 3′ and 5′ ends (e.g., via PCR) encompassing the variable region. The sequences of flanking adapters and bar-coding indices, if used, are specific to the sequencing technology; for review, see Blanco et al. (2020) and Newton et al. (2020). While a number of tools have been developed (Alam et al. 2015; Hannon 2010; Bolger et al. 2014; Martin 2011; Masella et al. 2012; Zhang et al. 2014; Aronesty 2013) to perform generic HTS DNA data pre-processing (e.g., trimming adapters, joining paired-end reads), these tools must be used in combination and are not customized for data characteristics of peptide selections, posing a barrier to entry for many biochemists. While tools such as FASTAptamer (Alam et al. 2015) or FASTX-Toolkit (Hannon 2010) can analyze nucleic acid selections, they lack functions related to peptides, including dereplication necessitated by degeneracy of the genetic code. Other tools, such as Enrich2 (Rubin et al. 2017) can analyze peptide selections; however, it requires the user to adhere to a strict experimental design, is not compatible with multi-lane sequencing runs, and is oriented toward computationally advanced users. The EasyDIVER pipeline (**Easy** pre-processing and **D**ereplication of **I**n **V**itro **E**volution **R**eads), described here, is a user-friendly, fast, one-step tool that can perform initial pre-processing, dereplication and translation (if desired) of Illumina sequencing data, suited for use with peptide or nucleic acid selections, with single or multi-lane sequencing runs. EasyDIVER enables the facile transition from raw sequencing data to processed data ready for a variety of downstream analyses. EasyDIVER also computes additional metrics to monitor the progress and success of the selection.

## EasyDIVER

**Fig. 1:**
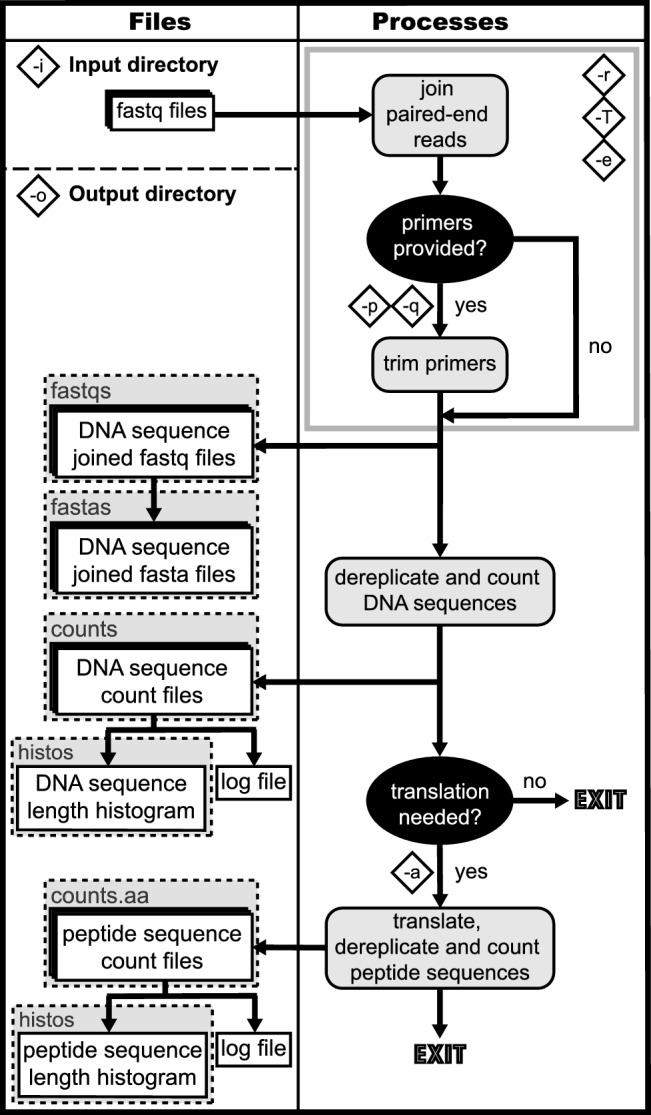
EasyDIVER flow chart. Input and output files are represented by white rectangles, subdirectory names by dashed gray rectangles, processes by rounded gray rectangles, and additional requirements by black ovals. Letters enclosed in diamond shapes represent flag variables (Table 1). The gray outline rectangle, together with the enclosed flags, represents the overall PANDAseq process

The EasyDIVER pipeline accepts as input raw, paired-end, demultiplexed Illumina read files (FASTQ) corresponding to multiple samples from one or more flow cell lanes (Fig. 1). The paired-end reads are joined using PANDAseq (Masella et al. 2012). The internal parameters used for PANDAseq can be customized in the pipeline. If forward and reverse primers are provided (at least one of them), assembled sequences are trimmed during the joining step using the user-supplied primer sequences. If the sequencing data correspond to reads from multiple lanes, reads from the different lanes are merged for each sample. For each provided sample (as well as for each individual lane, if desired), a dereplicated 'count' file of sequences is generated, listing all different sequences present in a sample and their absolute read counts and relative frequencies. For each sample (and optionally for the individual lanes), a text file with the sequence length distribution is generated.

For data corresponding to amino acid sequences (user-specified), sequences from the nucleotide count file are translated into amino acids and redundancies from translation are further dereplicated. Translation starts immediately after the user-specified extraction primer, or at the beginning of the sequence if no primers are provided. For each sample, a text file is generated with the length distribution of the amino acid sequences. Note that, if translation to amino acids is required, the user-specified forward extraction primer should be chosen such that the end of the primer sequence coincides with the end of a codon, ensuring the sequence is in frame after extraction (for more information see Supporting Figure S1 and Supporting Table S2).

Finally, a single log text file is created with information for each sample summarizing the progress of the process for a particular set of parameters values. An example of the text displayed in the Command Line Interface when running EasyDIVER can be found in Supporting Text S1.

### Requirements and Flag Variables

All input files must be in FASTQ format (either*.fastq* or compressed*.fastq.gz* extensions). Input files must follow the standard Illumina naming scheme: *sample-name_S#_L00#_R#_001.fastq*. EasyDIVER accepts a number of flag variables to control the parameters and options used in the process (Table 1). A more user-friendly alternative can be optionally used. If no flags are provided, the user will be prompted for input values in the command line in verbose form. It is worth noting that, although the prompted input version is seemingly easier to use, it has reduced versatility and its ability to be integrated in other pipelines is limited. An example of the text displayed in the Command Line Interface when running the prompted input version can be found in Supporting Text S2. For more information about the flag variables, see the Supporting Table S1 or the EasyDIVER manual, available at https://github.com/ichen-lab-ucsb/EasyDIVER.

**Table 1:** Flag variables

Flag	Description	Comments
-i	Input directory path and name	Required
-o	Output directory path and name	Optional Default value: /pipeline.output
-p	Extraction forward DNA primer	Optional
-q	Extraction reverse DNA primer	Optional
-T	Number of threads used for computation	Optional Default value: 1
-a	Translation into amino acids is performed	Optional Default value: FALSE
-r	Files for individual lanes are retained	Optional Default value: FALSE
-e	Additional internal PANDAseq flags	Optional Must be entered in quotation marks (e.g., -e “-L 50”) Default value: “-l 1 -d rbfkms“
-h	Help message	Optional

### Implementation

The pipeline script runs on Unix-based systems (e.g., Linux, Ubuntu, MacOS), via the Command Line Interface (often referred to as the Terminal). The translation step is performed by a Python script (translator.py), compatible with versions of Python 2 and Python 3. Source code and a test dataset (see “Results”) are freely available at https://github.com/ichen-lab-ucsb/EasyDIVER.

## Results

For each sample, the output files were redirected to the following sub-directories: *fastqs* (joined FASTQ files), *fastas* (joined FASTA files), *counts* (DNA counts files), *counts.aa* (peptide counts files), and *histos* (text files for length distributions). By default, the script suppresses output files from individual lanes (subdirectory *individual.lanes*).

We ran the pipeline using a test dataset from two samples of an experimental in vitro evolution of mRNA-displayed peptides (unpublished). The samples were sequenced by Illumina MiSeq (PE300), whose output was subsampled to give ~ 50,000 raw reads per sample. The library design for the test dataset is show in Supporting Figure S1.

**Fig. 2:**
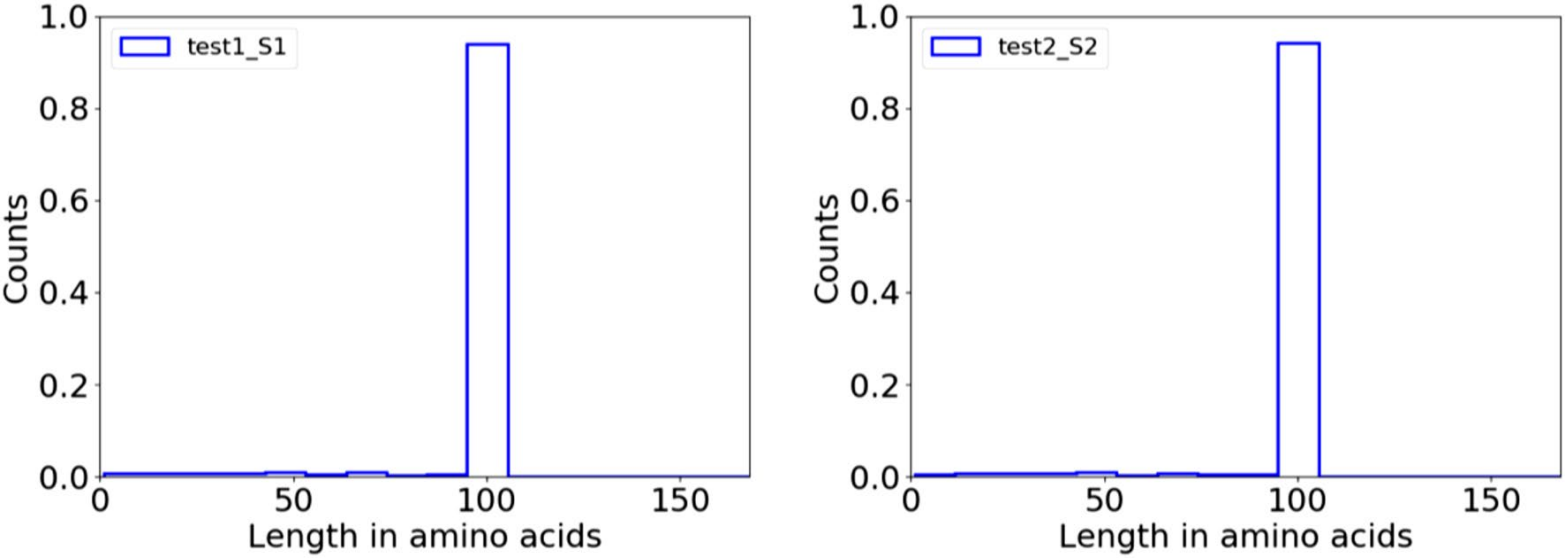
Peptide length histogram. Normalized length distribution of translated sequences for the two different samples in the test dataset: **a** test1_S1 and **b** test2_S2, using a bin size 10. See Supporting Figure S2 for the DNA length distributions

Additional information on the choice of input values can be found in Supporting Table S2. An example log text file is shown in Supporting Text S3. An example output peptide count file is shown in Supporting Text S4. Both samples conformed to the expected length distribution (97 amino acids corresponding to 291 nt; Fig. 2), and > 85% of the raw reads were recovered in DNA and peptide sequence count files.

EasyDIVER processed the test data in approximately 90 s using 14 threads on a 2.2 GHz Intel Core i7 (with 16 GB 1600 MHz DDR3 RAM), including peptide-level processing. The running time and memory usage increase linearly with the number of raw reads (assuming the same diversity distribution). The running time and memory usage are expected to depend on the pools’ sequence diversity.

## Limitations

EasyDIVER was designed for input files that follow the standard Illumina naming scheme and only handles data from paired-end reads. Trimming or filtering based on quality values was not implemented; if desired, the user should perform quality pre-treatment using other tools (Hannon 2010; Martin 2011; Schmieder and Edwards 2011) before applying EasyDIVER. Counting is performed using an *awk* command and cannot be parallelized. EasyDIVER does not correct for sense or antisense orientation; this should be accounted for in library preparation and sequencing. The sense strand should be sequenced as the forward read, and the antisense strand as the reverse read (if sequencing a paired-end library). Alternatively, the user can specify reverse complement primers to manually find the antisense orientations.

## Conclusion

Despite the obvious advantages of HTS, anecdotal evidence suggests that a lack of simple computational tools is a barrier for biochemists in this field. EasyDIVER is meant to lift this barrier by quickly producing counts files that can be easily understood and used for downstream analyses (e.g., multiple alignment or clustering). For samples containing ~ 10^6^ reads, the process is anticipated to take approximately 10–15 min (per sample) on a standard personal computer. A major advantage of EasyDIVER is the ability to run the pipeline locally; however, for samples of larger size, or for projects involving a high number of samples, utilizing non-local computing resources (e.g., remote servers) might be a better alternative.

## Electronic supplementary material

Below is the link to the electronic supplementary material.

Supplementary file1 (PDF 461 kb)

## Data Availability

Data are available upon request.

## Code Availability

Source code is freely available at https://github.com/ichen-lab-ucsb/EasyDIVER
