# Supplementary material for: EasyDIVER: A Pipeline for Assembling and Counting High-Throughput Sequencing Data from In Vitro Evolution of Nucleic Acids or Peptides
Source: J Mol Evol. 2020 Jun 11;88(6):477–81. doi: 10.1007/s00239-020-09954-0 (PMC7324411; doi:10.1007/s00239-020-09954-0)

Supporting Information

EasyDIVER: a pipeline for assembling and counting high throughput sequencing data from *in vitro* selections of nucleic acids or peptides

Celia Blanco, Samuel Verbanic, Burckhard Seelig and Irene A. Chen

---

Supporting Text S1. Command line interface output obtained from running EasyDIVER using the test data set: `easydiver.sh -i ./ -o ./output -p GGCGGAAAGCACATCTGC -T 14 -a -r`

```
raw.reads username$ easydiver.sh -i ./ -o ./output -p GGCGGAAAGCACATCTGC -T 14 -a -r
 ______                _____ _______      ________ _____
|  ____|              |  __ \_   _\ \    / /  ____|  __ \
| |__   __ _ ___ _   _| |  | || |  \ \  / /| |__  | |__) |
|  __| / _` / __| | | | |  | || |   \ \/ / |  __| |  _  /
| |____ (_| \__ \ |_| | |__| || |_   \  /  | |____| | \ \
|______\__,_|___/\__, |_____/_____|   \/   |______|_|  \_\
                  __/ |
                 |___/
+--------------------------------------------------------------------------------+
| Thu Jan 30 13:06:22 PST 2020                                                   |
|                                                                                |
| Welcome to the pipeline for Easy pre-processing and Dereplication of In Vitro  |
| Evolution Reads                                                                |
+--------------------------------------------------------------------------------+


-----Input directory path: /Users/username/Desktop/raw.reads
-----Output directory path: /Users/username/Desktop/raw.reads/output
-----Forward Primer: GGCGGAAAGCACATCTGC
-----No reverse primer supplied. Extraction will be skipped.
-----Number of threads = 14
-----No additional PANDAseq flags supplied.
-----Translation needed.
-----Individual lane outputs will be retained.

Input filecheck passed

Joining test1_S1_L001 reads & extracting primer...
Converting joined test1_S1_L001 FASTQ to FASTA...
Adding test1_S1_L001 reads to total test1_S1 reads...
Generating test1_S1_L001 nt length distribution for individual lanes...
Calculating unique & total reads for lane test1_S1_L001...
Collecting unique, total and sequences in file...

Joining test2_S2_L001 reads & extracting primer...
Converting joined test2_S2_L001 FASTQ to FASTA...
Adding test2_S2_L001 reads to total test2_S2 reads...
Generating test2_S2_L001 nt length distribution for individual lanes...
Calculating unique & total reads for lane test2_S2_L001...
Collecting unique, total and sequences in file...

Calculating unique & total reads for test1_S1...
Calculating unique & total reads for test2_S2...

Individual lane outputs will be retained

Generating test1_S1 DNA length distribution...
Translating test1_S1 DNA to peptides...
Generating test1_S1 aa length distribution...

Generating test2_S2 DNA length distribution...
Translating test2_S2 DNA to peptides...
Generating test2_S2 aa length distribution...

Run time: 87
```

Supporting Text S2. Command line interface output obtained from running EasyDIVER using the prompted input version (no flags provided): `easydiver.sh`. The rest of the output has been omitted for the sake of simplicity (see Supp. Text S1).

```
raw.reads username$ easydiver.sh
 ______                 _____ _______      ________ _____
|  ____|               |  __ \_   _\ \    / /  ____|  __ \
| |__   __ _ ___ _   _ | |  | || |  \ \  / /| |__  | |__) |
|  __| / _` / __| | | || |  | || |   \ \/ / |  __| |  _  /
| |___| (_| \__ \ |_| || |__| || |_   \  /  | |____| | \ \
|______\__,_|___/\__, ||_____/_____|   \/   |______|_|  \_\
                  __/ |
                 |___/
+----------------------------------------------------------------------------------+
| Thu May 22 17:11:15 PST 2020                                                     |
|                                                                                  |
| Welcome to the pipeline for Easy pre-processing and Dereplication of In Vitro    |
| Evolution Reads                                                                  |
+----------------------------------------------------------------------------------+

NO FLAGS PROVIDED. ENTERING PROMPTED INPUT VERSION

Path to your input directory:
./

Path to your output directory (default value /pipeline.output):
./output

Forward primer sequence for extraction:
GGCGGAAAGCACATCTGC

Reverse primer sequence for extraction:


Number of threads desired for computation (default value 1):
14

Extra flags for PANDAseq (default value "-l 1 -d rbfkms"; see manual):


Perform translation into amino acids? (yes / no)
yes

Retain output files for individual lanes? (yes / no)
yes

-----Input directory path: /Users/username/Desktop/raw.reads
-----Output directory path: /Users/username/Desktop/raw.reads/output
-----Forward Primer: GGCGGAAAGCACATCTGC
-----No reverse primer supplied. Extraction will be skipped.
-----Number of threads = 14
-----No additional PANDAseq flags supplied.
-----Translation needed.
-----Individual lane outputs will be retained.

Continues …
```

Supporting Table S1. Flag variables. Extended information and consideration in the use of flag variables.

Flag and value	Comments
-i input.directory	Required. Input directory path and name. If no value is provided, an error message will be printed in the terminal: `ERROR: No input filepath supplied` and no further action will be performed.
-o output.directory	Optional. Output directory path and name. If no value is provided, the default value `/pipeline.output` will be used.
-p forward.primer	Optional. Extraction forward DNA primer. If a forward primer sequence is provided, the pipeline strips out the primer from the start of the sequence. Any sequence before the provided primer will be discarded.
-q reverse.primer	Optional. Extraction reverse DNA primer. If a reverse primer sequence is provided, the pipeline strips out the primer at the start of the sequence. Any sequence after the provided primer will be discarded.
-T threads	Optional. Number of threads used for computation. Default value is 1. The number of threads that may be used is dependent on the user's CPU (for example, if using a machine with 16 threads, `-T 14` could be a desirable number). The default value of 1 would be suboptimal for multi-core machines.
-a	Optional. Translation into amino acids is performed. DNA sequences are translated using the standard genetic code, and the resulting sequences are dereplicated. By default, translation is not performed.
-r	Optional. Files for individual lanes are retained. By default, the script will suppress outputs from individual lanes.
-e	Optional. Additional internal PANDASeq flags. Values must be entered in quotation marks (e.g. `-e "-L 50"`). Default value is `"-l 1 -d rbfkms"`. For more information see the PANDASeq manual*.
-h	If used, a help message will be printed in the terminal and no further action will be performed.

* Masella, A. P., Bartram, A. K., Truszkowski, J. M., Brown, D. G. & Neufeld, J. D. 2012. PANDAseq: paired-end assembler for illumina sequences. *BMC Bioinformatics,* 13**,** 31.

Supporting Figure S1. Library design for the test dataset. Test dataset from two samples of an experimental *in vitro* evolution of mRNA-displayed peptides (unpublished).

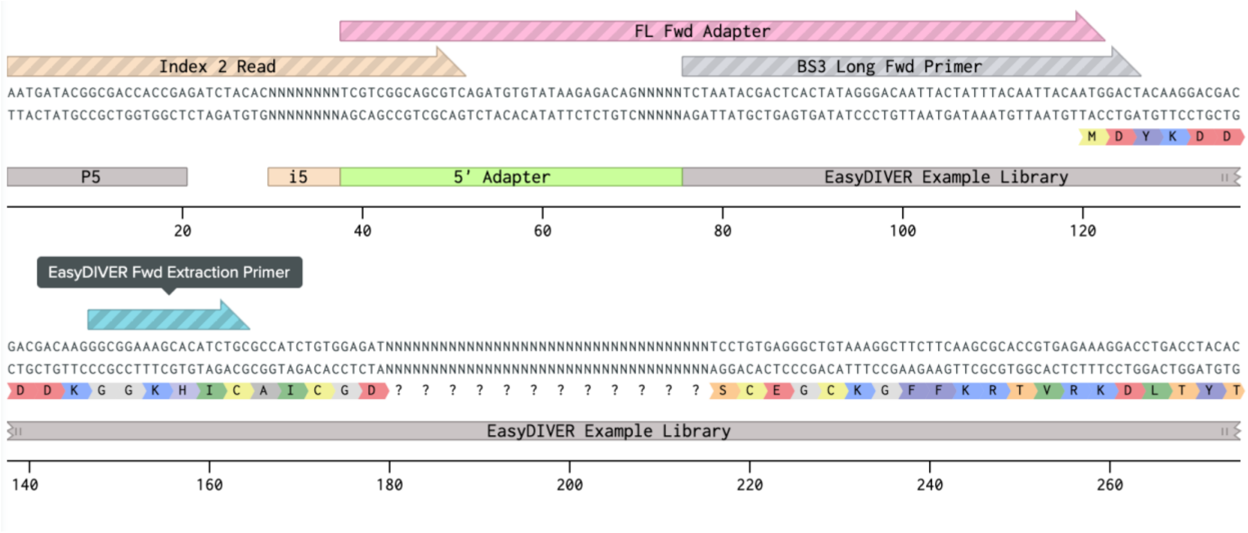

Supporting Table S2. Choice of input values. Explanation of the choice of input values for the example provided in Supporting Text S1 and S2.

Extraction forward DNA primer	**Note:** The choice of primers determines how the target sequences are extracted and set the reading frame for translation. Extraction primers should target conserved sequences in the library and place the extracted sequences in the desired reading frame<br><br>**Example:** In the example command provided in Supp. Table S1 and Supp. Table S2, the primer GGCGGAAAGCACATCTGC corresponds to a conserved portion of the library, and should be present in every sequence. The extracted sequence will be in the desired reading frame, starting with the amino acids AICGD, followed by the random portion of the library.
Number of threads used for computation	**Note:** Modern processors possess the capability to run processes in parallel by using multiple threads. Certain processes in EasyDIVER (such as joining with PANDAseq) can utilize this capability to run faster. For optimal performance, the number of threads should not exceed the number of cores on the machine. For further optimization, the hardware specifications should be considered and the number of threads adjusted accordingly.<br><br>**Example:** In the example command provided in Supp. Table S1 and Supp. Table S2, the thread count is set as -T 14, which would be a desirable choice for a machine with 16 threads. To be safe, and assuming the machine used is at least a quad-core CPU, a maximum of 4 threads should be used.

Supporting Text S3. Results displayed in the file log.txt, obtained from running EasyDIVER using the provided test dataset. Executed from the local directory raw.reads, using the flags `-i ./ -o ./output -p GGCGGAAAGCACATCTGC -T 14 -a`.

-----Input directory path: /Users/username/Desktop/raw.reads

-----Output directory path: /Users/username/Desktop/raw.reads/output

-----Forward Primer: GGCGGAAAGCACATCTGC

-----Individual lane outputs suppressed

-----Number of threads = 14

-----Translation on

-----No additional PANDAseq flags

sample	fastq_R1	fastq_R2	unique_nt	total_nt	recovered_nt(%)	unique_aa	total_aa	recovered_aa(%)
test1_S1	64516	64516	54168	55576	86.14%	38695	55556	86.11%
test2_S2	53541	53541	45131	46605	87.05%	31147	46593	87.02%

Supporting Text S4. Partial peptide count file for sample test1_S1 (test1_S1_counts.aa.txt). Excerpt shows 10 most abundant different sequences present in the sample and their absolute read counts and relative frequencies.

```
number of unique sequences = 38695

total number of molecules = 55556


AICGDVVATADTKIQYDSCEGCKGFSKRTVRKDLTYTCRDYKDCECYHKCLDLCQYCRYQKALAMGMKREAVQEEVGSHHQHHHGGSMGMSGSGTGY     2189  3.940%

AICGDYISAVDTQSKNDSCEGCKGFFKRTVRKDLTYTCRDNKNCECYHFCLQNCQYCRYQKALAMGMKREAVQEEVGSHHHHHHGGSMGMSGSGTGY     847   1.525%

AICGDYISAVDTQSKNDSCEGCKGFFKRTVRKDLTYTCRDNKDCECYHFCLQNCQYCRYQKALAMGMKREAVQEEVGSHHQHHHGGSMGMSGSGTGY     682   1.228%

AICGDVVATADTKIQYDSCEGCKGFSKRTVRKDLTYTCRDYKDCESYHKCSDLCQYCRYQKALAMGMKREAVQEEVGSHHQHHHGGSMGMSGSGTGY     589   1.060%

AICGDVVATADTKIQYDSCEGCKGFSKRTVRKDLTYTCRDYKDCECYHKCLDLCQYCRYQKALAMGMKRKAVQEEVGSHHQHHHGGSMGMSGSGTGY     511   0.920%

AICGDYISAVDTQSKNDSCEGCKGFFKRTVRKDLTYTCRDNKNCECYHFCLQNCQYCRYQKALAMGMKREAVQEEVGSHHQHHHGGSMGMSGSGTGY     328   0.590%

AICGDVVATADTKIQYDSCEGCKGFSKRTVRKDLTYTCRDYKNCESYHKCLDLCQYCRYQKALAMGMKREAVQEEVGSHHQHHHGGSMGMSGSGTGY     267   0.481%

AICGDVVATADTKIQYDSCEGCKGFSKRTVRKDLTYTCRDYKDCECYHKCLDLCQYCRYQKALAMGMKREAVQEEVGSHHQPHHGGSMGMSGSGTGY     264   0.475%

AICGDYISAVDTQSKNDSCEGCKGFFKRTVRKDLTYTCRDNKDCECYHFCLQNCQYCRYQKALAMGMKREAVQEEVGSHHHHHHGGSMGMSGSGTGY     224   0.403%

AICGDYISAVDTQSKNDSCEGCKGFFKRTVRKDLTNTCRDNKNCECYHFCLQNCQYCRYQKALAMGMKREAVQEEVGSHHHHHHGGSMGMSGSGTGY     215   0.387%
```

Supporting Figure S2. DNA sequence length histogram. Normalized length distribution of DNA sequences for the two different samples (test1_S1 and test2_S2) in the test dataset (bin size 10). Expected length is 291 nt.

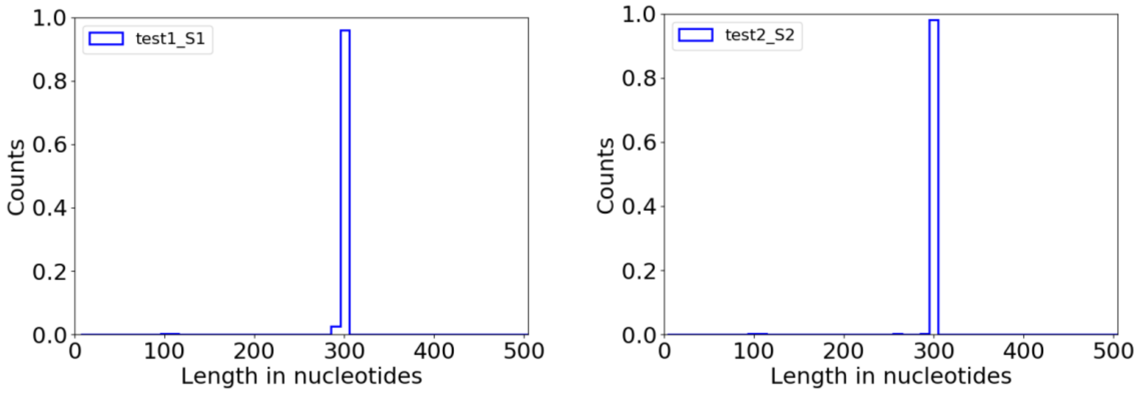

Supplement: Supplementary file 1 — Supplementary file1 (PDF 461 kb) [file 239_2020_9954_MOESM1_ESM.pdf]
